# Role of Lipiodol® lymphangiography in the diagnosis and management of post-operative chylous ascites

**DOI:** 10.3389/fradi.2025.1537744

**Published:** 2025-06-02

**Authors:** Alexis Litchinko, E. Monnard, C. Tappero, B. Egger

**Affiliations:** ^1^ Division of Digestive Surgery, Fribourg Cantonal Hospital, Fribourg, Switzerland; ^2^ Division of Digestive Surgery, University Hospitals of Geneva, Genève, Switzerland; ^3^ Division of Radiology, Fribourg Cantonal Hospital, Fribourg, Switzerland

**Keywords:** Lipiodol®, chylous ascites, chylous, surgery, leakage

## Abstract

**Introduction:**

Chylous ascites, defined as the pathological accumulation of lymphatic fluid in the peritoneal cavity, presents significant challenges due to the lack of standardized treatment protocols. This study evaluates the dual diagnostic and therapeutic role of Lipiodol® lymphangiography in managing post-operative chylous ascites, with a focus on its potential to inform modern interventional strategies.

**Materials and methods:**

A retrospective review was conducted of four patients treated for post-operative chylous ascites at our institution between 2017 and 2023. These patients, who developed refractory chylous ascites following oncological surgeries involving radical lymphadenectomy, underwent Lipiodol® lymphangiography. Diagnostic findings, therapeutic outcomes, and procedural details were analyzed.

**Results:**

Lipiodol® lymphangiography demonstrated a dual function, providing precise anatomical localization of lymphatic leaks while facilitating therapeutic embolization due to its viscosity. All four patients achieved resolution of chylous ascites following lymphangiography alone, with a 100% success rate after the first attempt. No complications were reported within 30 days post-procedure, underscoring the safety of this minimally invasive technique.

**Conclusions:**

Lipiodol® lymphangiography represents an effective diagnostic and therapeutic modality for post-operative chylous ascites, offering a minimally invasive alternative to traditional surgical interventions. By elucidating the pathway for both diagnosis and treatment, this study highlights its potential role in establishing standardized protocols for managing this complex condition.

## 1 Introduction

Post-operative chylous ascites is a rare but distressing complication that can occur following various surgical procedures, particularly thoraco-abdominal surgeries (1–6). It is characterized by the accumulation of milky, chyle-rich fluid within the peritoneal cavity, leading to significant morbidity and prolonged hospital stays. While its exact incidence remains uncertain, chylous ascites poses a considerable challenge for both patients and surgeons due to its complex etiology and limited treatment options (7, 8).

The leakage of chyle into the peritoneal cavity disrupts fluid balance and leads to the loss of essential proteins, resulting in hypoalbuminemia. This imbalance affects fluid homeostasis and can cause peripheral edema. Additionally, the continuous loss of chyle can lead to nutritional deficiencies, particularly in fat-soluble vitamins (vitamins A, D, E, and K), impacting wound healing, immune function, and overall post-operative recovery. Electrolyte imbalances may occur due to the loss of electrolytes such as sodium, potassium, and calcium along with the chyle, potentially affecting nerve conduction, muscle function (including cardiac and musculoskeletal), and fluid balance. Furthermore, chyloperitoneum can impair immune function, increasing the risk of post-operative infections and major complications (2, 5). Severe cases can cause increased intra-abdominal pressure, potentially leading to respiratory distress, reduced lung capacity, and abdominal compartment syndrome (9, 10).

Managing the physiological consequences of post-operative chyloperitoneum requires a comprehensive approach. Treatment strategies aim to address the underlying cause, restore fluid and protein balance, provide adequate parenteral nutrition, correct electrolyte imbalances, and support immune function if necessary (11). Conservative measures such as strict dietary modifications and medium-chain triglycerides may be employed initially. However, persistent or severe chyloperitoneum may require more invasive interventions, including surgical repair of lymphatic leaks or embolization procedures to control chyle leakage and mitigate the associated physiological consequences (10). Close monitoring, assessment of nutritional status, electrolyte levels, and fluid balance, and appropriate interventions are crucial in optimizing recovery and improving patient outcomes. The complexity of chylous ascites management requires an integrated approach that combines precise diagnostic techniques with effective therapeutic strategies, minimizing patient morbidity and resource utilization.

Although conservative measures such as dietary modifications or total parenteral nutrition can be effective in some cases, they often fail to resolve persistent chyle leaks. In this context, lymphangiography has emerged as a pivotal technique, not only for identifying lymphatic leaks with precision but also for its inherent embolization effect, which can address leaks directly (12–17). Traditionally, the diagnosis of chylous ascites has relied on clinical signs, laboratory investigations, and imaging studies such as ultrasonography and computed tomography (CT). While traditionally considered a diagnostic tool, lymphangiography has gained attention for its therapeutic potential in addressing lymphatic leaks. This dual role highlights its value in minimizing the need for more invasive interventions. However, these modalities often lack the specificity and precision necessary to accurately identify the underlying cause and guide appropriate treatment decisions.

Lymphangiography, also known as contrast-enhanced lymphography, involves the injection of contrast material into the lymphatic system followed by imaging to visualize the lymphatic vessels and identify abnormalities. Initially used primarily for lymphatic mapping in cancer staging, lymphangiography has gained attention for its potential role in the evaluation and management of chylous ascites. By providing detailed anatomical information about the lymphatic system, this technique offers valuable insights into the site of lymphatic leakage and aids in planning targeted interventions (8, 13, 16).

This study explores the utility of Lipiodol® lymphangiography in bridging the gap between diagnosis and treatment of post-operative chylous ascites, aiming to establish its role as a minimally invasive alternative to traditional surgical approaches. We discuss the diagnostic challenges associated with this condition and highlight the advantages of lymphangiography over conventional imaging techniques. Furthermore, we delve into the various treatment options available for chylous ascites and explore how lymphangiography can be utilized to guide therapeutic interventions, including the application of embolization techniques.

Through this case series describing our centre's experience in managing post-operative chyloperitoneum, we aim to provide clinicians and researchers with a deeper understanding of lymphangiography's role in the management of post-operative chylous ascites. By shedding light on this emerging field, we hope to facilitate improved patient outcomes, minimize unnecessary interventions, and pave the way for further advancements in the diagnosis and treatment of this challenging condition.

It is crucial to acknowledge that while lymphangiography shows promise, its implementation and clinical effectiveness may vary across different healthcare settings. Therefore, ongoing research and collaborative efforts are necessary to refine protocols, establish guidelines, and ascertain its optimal utilization in the management of post-operative chylous ascites.

## 2 Materials and methods

### 2.1 Patient characteristics

To identify patients who underwent lymphangiography for chylous ascites, we conducted a retrospective analysis of databases at our institutions from January 2017 to June 2023. Institutional Review Board approval was obtained for this retrospective study focusing on percutaneous intervention for lymphatic disorders. Information regarding patient characteristics, treatment approaches, and follow-up was extracted from the electronic medical records as outlined in Table 1. All participants provided their written consent, and the study received approval from the regional ethics board, recorded with the BASEC-ID 2021-02526. In total, four patients with chylous ascites were treated with lymphangiography.

**Table 1:** Demographics and clinical details of included patients before embolization.

Patient ID	Gender	Age	Type of surgical procedure	Duration of ascites (days)	Average volume per day (ml)
1	F	66	Whipple procedure	7	250–350
2	F	81	Whipple procedure	9	350–400
3	F	61	Whipple procedure	9	300–400
4	F	54	Laparoscopic right hemicolectomy	11	250–400

### 2.2 Definition of chylous leakage

Chylous ascites or chylous leakage is defined as a pathological extravasation of chyle, a triglyceride-rich lymphatic fluid, into abdominal cavities or post operative sites typically resulting from injury to the intestinal lymphatics or lymphatic trunks below the cisterna chyli; the cause of such a leakage is various. They can range from a lesion of the lymphatic ducts (traumatic or post-operative) to a cancerous infiltration. It can be biologically defined as a milky fluid composed primarily of lymph and emulsified fats (triglycerides), in body fluids collected from cavities or surgical drains. Diagnostic confirmation is typically achieved mostly clinically but can be through laboratory analysis, revealing elevated triglyceride levels, often exceeding 110 mg/dl, and the presence of chylomicrons in the fluid sample with specific analysis. In this studies, chylous leakage has been clinically suspected and then all confirmed biologically.

### 2.3 Lymphangiography technique

The conventional method used in lymphatic vessel mapping hinges on the cannulation of a lymphatic vessel located in the foot. However, for our specific cases, the primary interest was the abdominal lymphatic vessel. As a result, we opted for the technique delineated by Nadolski and Itkin in 2012, injecting the contrast in the inguinal region (18). In all procedures, a 22G BD Nexiva Diffusics was used for percutaneous access to the inguinal lymph nodes. The needle selection aimed to provide optimal penetration while minimizing tissue trauma. This method stands out for its precision and efficacy in targeting the abdominal region and is a much easier process compared to the conventional method (19, 20).

Guided by ultrasound, a 20 G needle is meticulously inserted into the cortex of a lymph node situated in the groin area on both sides. To ascertain the needle's correct positioning, an ultrasound contrast medium, specifically SonoVue® (manufactured by Bracco Suisse SA), was administered. A correctly positioned needle is indicated by the retention of the contrast medium solely within the lymph node. This verification step is vital because it prevents the inadvertent injection of the contrast medium into the surrounding soft tissue or, even worse, directly into the vascular circulation. Such an error could lead to grave complications, including the potential for pulmonary embolism and in presence of PFO (Patent Foramen Ovale) also to CVA (cerebrovascular accident).

**Figure 1:**
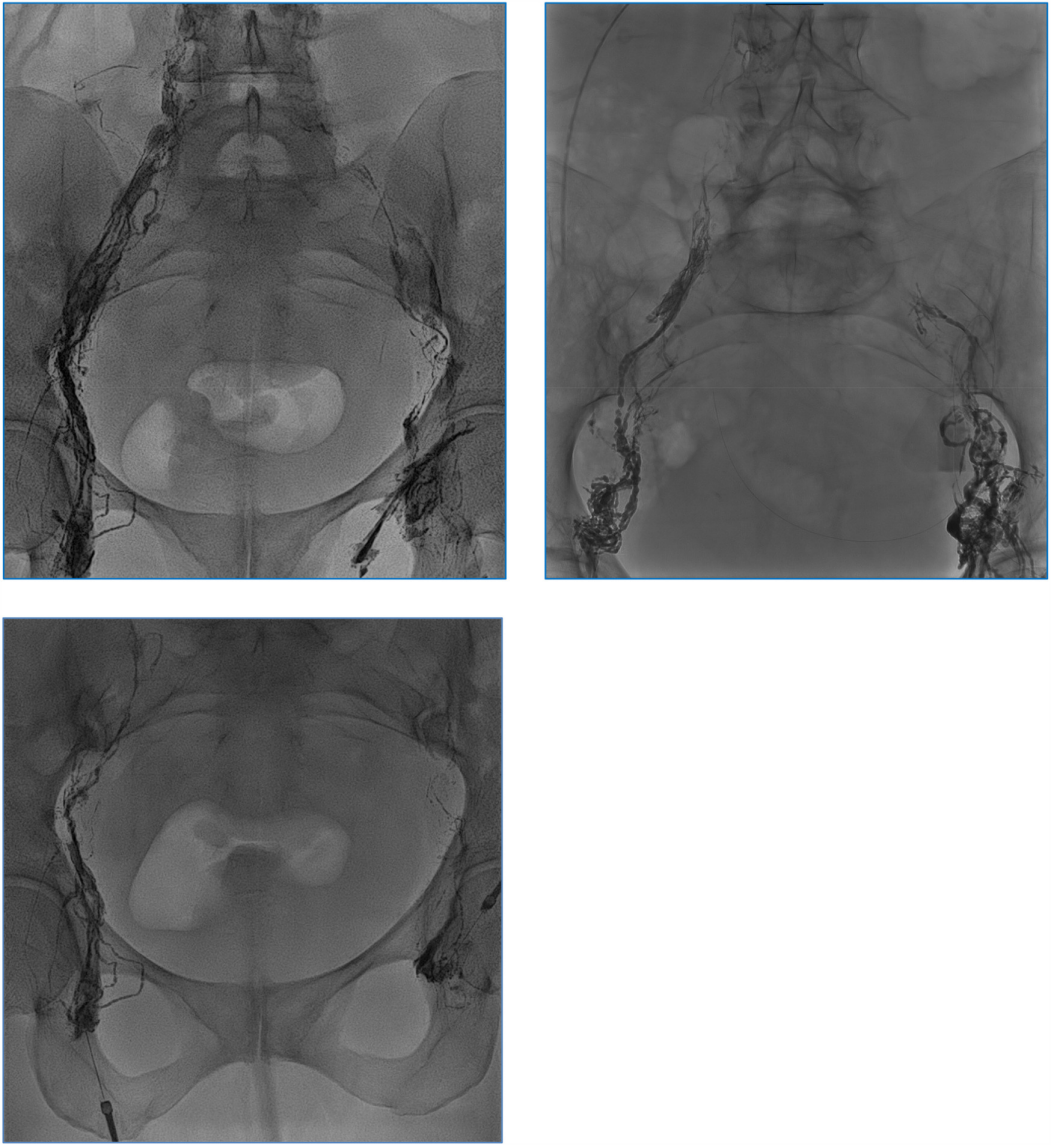
Opacification of the iliac lymphatic ducts during injection of ethiodized oil (Lipiodol®). No leakage was depictable.

**Figure 2:**
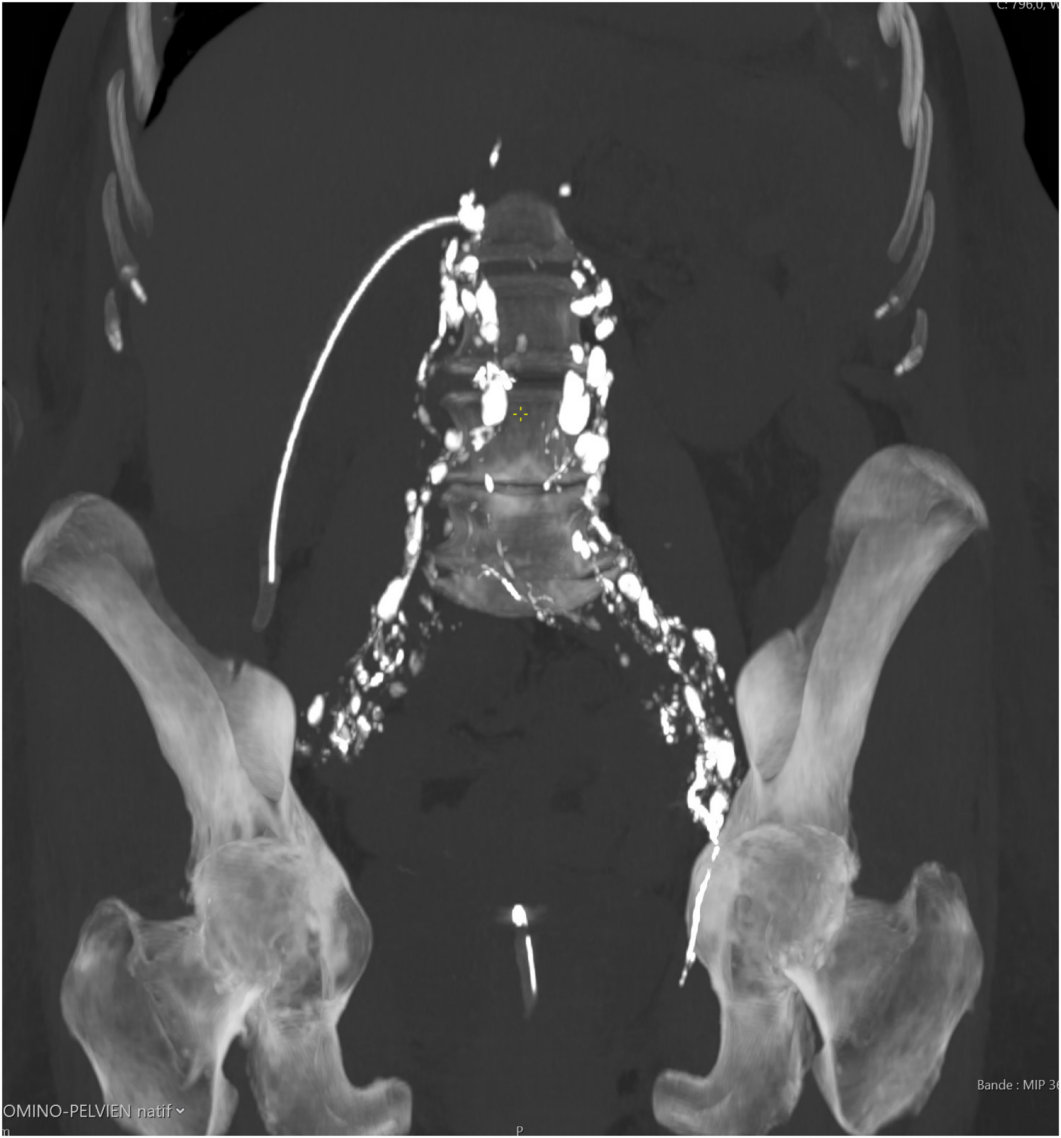
Axial CT scan after intranodal Lipiodol® demonstrating dense opacification of pelvic and paraaortic lymphatics without evidence of leakage.

Once the needle's positioning is confirmed as accurate, it is securely anchored to the skin. Following this, Lipiodol® Ultra Fluid (produced by Guerbet AG) is injected at a regulated pace of 1 ml every 10 min, all while under the vigilant eye of fluoroscopy (Figure 1). Typically, the injection volume does not exceed 10 ml of Lipiodol for each lymph node. All procedures were monitored by fluoroscopy using a Siemens Artis Zee system, and Lipiodol® Ultra Fluid (Guerbet AG) was injected manually via a 1 ml syringe at 10-minute intervals. The maximum volume injected per lymph node was limited to 10 ml. If no leak was detected on fluoroscopy, a non-contrast-enhanced CT scan was performed within 1 h to track Lipiodol® migration and identify occult leaks. In our series, CT was used in all four patients and did not result in any change of management. If any leakage of the contrast medium remained undetected during the fluoroscopy phase, we proceeded with non-contrast CT scans. These scans enabled us to monitor the movement of the contrast medium through the lymphatic vessel meticulously and detect even minute leakages, assisting in determining the most effective subsequent intervention. In all cases, post-procedural non-contrast-enhanced CT scans were obtained to assess Lipiodol® migration and exclude occult leakage. Figure 2 illustrates a typical example of such distribution, confirming adequate opacification of the abdominal lymphatic vessels.

**Figure 4:**
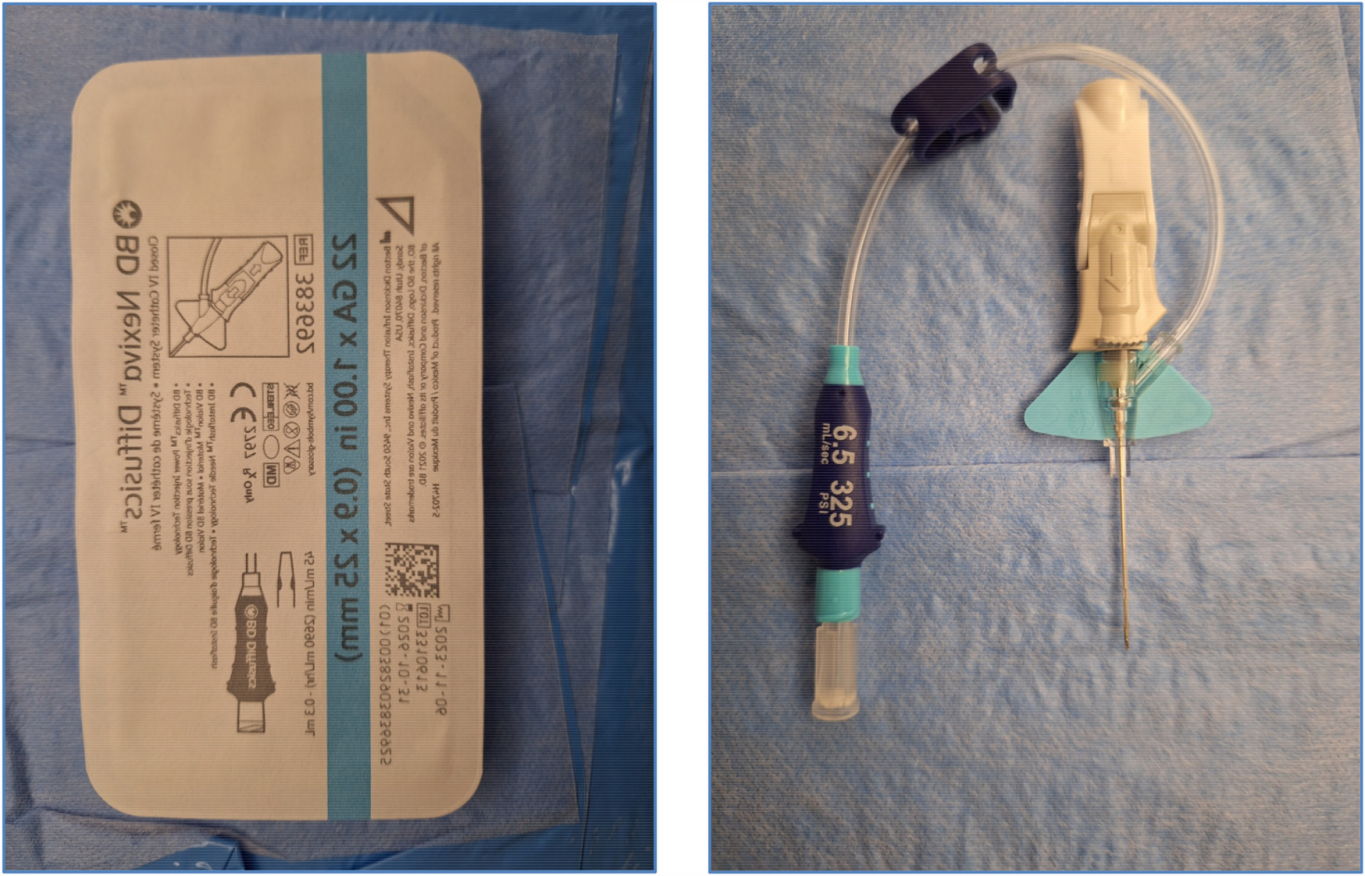
22G BD nexiva Diffusics® catheter.

**Figure 5:**
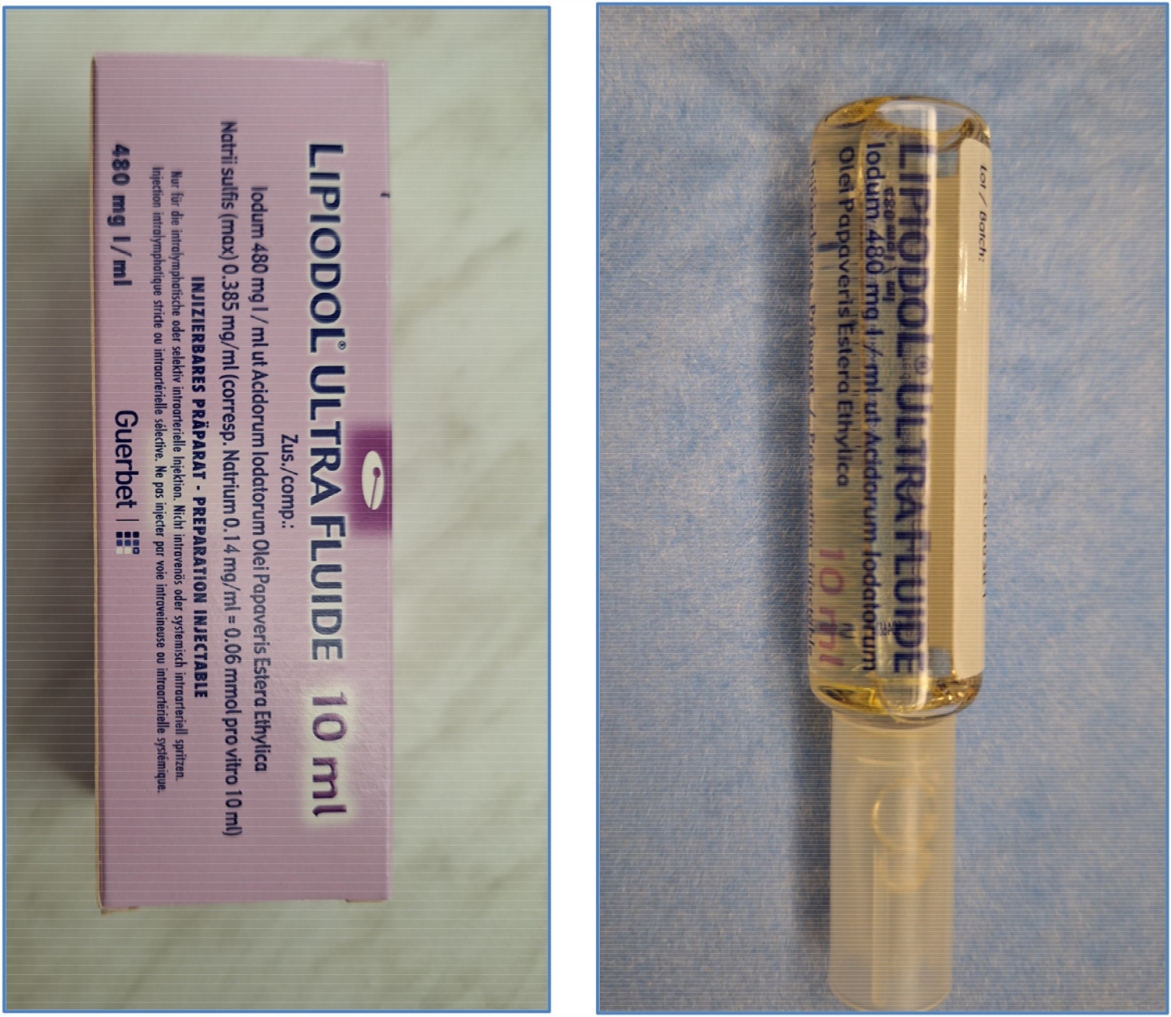
Lipiodol® ultra fluid used during the procedure.

### 2.4 Embolisation technique

In our series, no embolization procedure was performed, as no active lymphatic leak was visualized during fluoroscopy or subsequent CT imaging. Therefore, no microcatheters, guidewires, or embolic materials (such as glue or coils) were used.

The intervention relied exclusively on the injection of Lipiodol® via the 22G BD Nexiva Diffusics® catheter (Figures 4, 5), under fluoroscopic guidance. The ethiodized oil was monitored in real time to assess its distribution through the lymphatic system and detect potential leak sites. This approach, while technically simplified, aimed to harness the inherent pro-inflammatory and embolic properties of Lipiodol® to promote spontaneous leak closure. In cases requiring embolization, our institutional protocol allows for escalation to more advanced techniques, including microcatheter-based embolization, performed by an interventional radiologist. However, such steps were not necessary in this series, as all patients responded favorably to lymphangiography alone. While precautions were taken to avoid inadvertent intravascular injection, it is acknowledged that some degree of systemic migration of Lipiodol®, including pulmonary and cerebral distribution, may occur due to the lymphatic drainage into the venous system.

## 3 Results

### 3.1 Demographic data

We retrospectively reviewed four patients during the inclusion period. Table 1 summarizes the demographic and clinical details of the included patients. The patients, all female, vary in age from 54 to 81 years. The surgical procedures include three instances of the Whipple procedure, performed on patients aged 66, 81, and 61 and one laparoscopic right hemicolectomy performed on a 54-year-old patient.

The duration of ascites among these patients ranges from 7 to 11 days. Specifically, the patient aged 66 experienced ascites for 7 days, while the patients aged 81 and 61 each experienced it for 9 days. The patient who underwent the laparoscopic right hemicolectomy experienced the longest duration of ascites at 11 days.

The volume of ascitic fluid produced daily by these patients also varies. The patient aged 66 had a daily ascitic fluid volume ranging between 250 and 350 millilitres. The 81-year-old patient had a higher daily volume of 350 to 400 millilitres. The patient aged 61 produced between 300 and 400 millilitres of ascitic fluid per day, while the patient aged 54, who had the laparoscopic right hemicolectomy, produced between 250 and 400 millilitres daily.

### 3.2 Evolution of chylous ascites

In all four cases, resolution of chylous ascites was achieved following a single session of Lipiodol® lymphangiography without requiring embolization. No contrast leakage was identified on fluoroscopy or CT. This corresponds to a 100% technical and clinical success rate in our series, acknowledging the limitations of a small cohort. The viscosity of Lipiodol® likely contributed to embolization, explaining this high success rate. Importantly, no complications were reported within 30 days post-procedure. As no active lymphatic leak was visualized in any of the four cases, embolization was not performed. The therapeutic benefit was attributed to the inflammatory and viscous properties of Lipiodol® alone.

**Figure 3:**
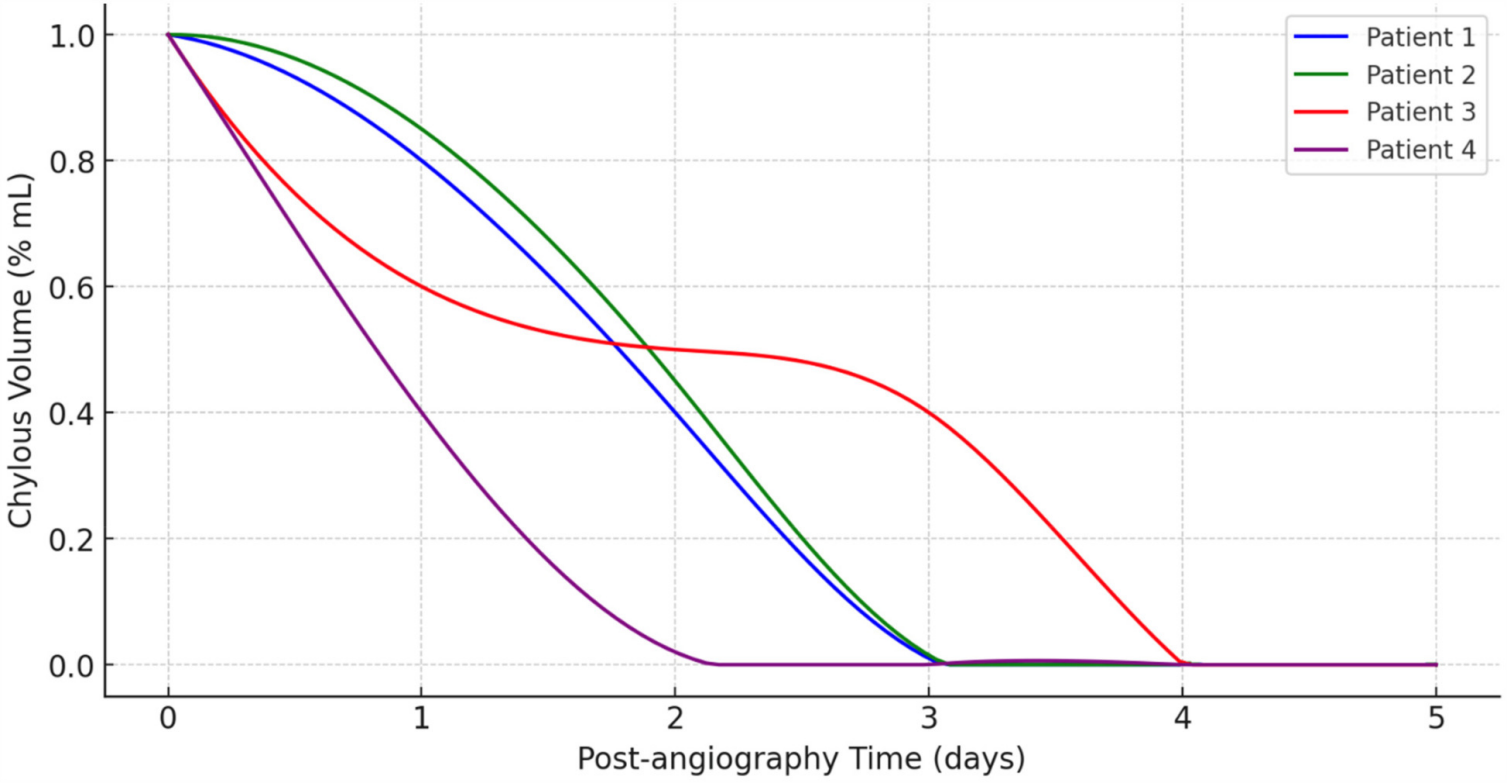
Reduction in daily chylous output over five days following Lipiodol® lymphangiography.

Evolution of chylous volume over time after lymphangiography is detailed in Figure 3. It depicts the reduction in chylous volume (% ml) over a span of five days following lymphangiography of all patients. The *x*-axis represents the post-lymphangiography time in days, while the *y*-axis shows the chylous volume as a percentage of the initial volume.

Initially, all four patients start with a chylous volume at 100% immediately after angiography (Day 0). Patient 4, represented by the purple line, exhibits the most rapid decrease in chylous volume, reaching near-zero levels by Day 1. Patient 1 (blue line) and Patient 2 (green line) show a similar trend, with their chylous volume decreasing steadily and reaching zero around Day 3. Patient 3, depicted by the red line, shows a slower reduction compared to the others. This patient's chylous volume declines gradually, not reaching near-zero levels until around Day 4. Notably, Patient 3 has a more prolonged period with a significant volume of chyle compared to the other patients.

Overall, this graph (Figure 1) demonstrates that lymphangiography is effective in reducing chylous volume in post-operative patients, with all patients showing a significant decrease within a few days. However, the rate of reduction varies among patients, indicating that individual responses to the procedure can differ.

### 3.3 Pre-lymphangiography management

Before proceeding to lymphangiography, all patients underwent an initial course of conservative management. Dietary modifications were systematically implemented, including a low-fat regimen enriched with medium-chain triglycerides (MCTs) to reduce chyle flow via the intestinal lymphatics. In two cases, total parenteral nutrition (TPN) was introduced after failure of enteral strategies, allowing for bowel rest and further minimizing lymphatic output. Pharmacologic therapy with subcutaneous octreotide was initiated in three patients to decrease splanchnic circulation and inhibit lymph production. Despite these interventions, chyle output remained persistently high—ranging between 250 and 400 ml/day—which prompted the indication for interventional lymphangiography. The median duration of conservative treatment prior to the procedure was 7 days (range: 5–10 days).

### 3.4 Other management techniques

More traditional approaches for managing post-operative chylous ascites include conservative, pharmacological, and surgical interventions (3, 21, 22). Conservative treatment involves dietary modifications and supportive care to reduce chyle production and promote its reabsorption. Patients are often placed on a low-fat diet supplemented with medium-chain triglycerides (MCTs), which are absorbed directly into the portal venous system, bypassing the lymphatic system. In severe cases, total parenteral nutrition (TPN) may be used to rest the gastrointestinal tract entirely and minimize lymph flow. Pharmacological interventions can also be employed, with somatostatin and its analog octreotide commonly used to decrease lymph production by reducing splanchnic blood flow and inhibiting the release of gastrointestinal hormones. Diuretics may be used to manage fluid balance and reduce ascitic fluid volume, though they do not address the underlying lymphatic leak. When conservative and pharmacological treatments are ineffective, surgical options may be considered. Surgical interventions aim to directly address the lymphatic leak or divert the chyle flow and include techniques such as direct ligation or suturing of leaking lymphatic vessels during re-exploration surgery, the creation of a peritoneovenous shunt to redirect chyle into the venous system, and lymphaticovenous anastomosis to establish a new drainage pathway for lymphatic fluid (23–25). These surgical procedures are typically reserved for cases where non-invasive measures have failed and require careful consideration of the risks and benefits. Each of these management techniques has its own indications, advantages, and potential complications, and the choice of treatment depends on the severity of the chylous ascites, the patient's overall condition, and the underlying cause of the lymphatic leak. Combining different approaches may sometimes be necessary to achieve optimal outcomes.

## 4 Discussion

This comprehensive investigation emphasizes the crucial role and effectiveness of lymphangiography, particularly intranodal ethiodized oil injection, in managing chylous leaks following abdominal surgery. The study strongly supports the high technical success rate of this method, aligning with previous research in theragnostic lymphangiography for treating pelvic and abdominal leaks (26–29). Significantly, the safety of this procedure is underscored by the absence of major lymphography-related complications despite the administration of ethiodized oil volumes exceeding those reported in other series. The therapeutic effect of Lipiodol® is believed to stem from its pro-inflammatory properties, leading to occlusion of small lymphatic vessels at the leakage site. This embolizing potential, described in multiple series, supports its use as a first-line intervention, especially when a visible leak is not evident.

Our retrospective analysis reveals that patients who underwent lymphangiography had notably shorter treatment durations for chylous leakage compared to those who did not receive this intervention. This finding is critically important as it indicates the potential of lymphangiography not only as a treatment for persistent or refractory cases but also as an early proactive measure in the management of chylous leakage. The accelerated recovery observed in patients undergoing lymphangiography and, if necessary embolisation, points towards its effectiveness in hastening the healing process, potentially reducing hospital stays and improving patient outcomes.

Furthermore, our study sheds light on predictive factors for the development of chylous leakage following abdominal surgery. These include the type of abdominal surgery (particularly for malignant conditions), the number of harvested lymph nodes, and the early postoperative drain fluid volume. These insights are vital for understanding the risk factors associated with chylous leakage, highlighting the complex interplay of surgical variables in its development. Identifying these predictive factors is key to devising more targeted prevention and management strategies for chylous leakage in abdominal surgery patients (5, 10, 30–32). The analysis also brings into focus the unique challenges and considerations in managing postsurgical lymphatic leaks. Literature suggests that multiple sessions of lymphangiography might be necessary to achieve clinical success. This iterative approach, while requiring additional procedures, underlines the need to tailor the treatment to individual patient conditions and the dynamic nature of lymphatic leak management (33–36).

However, the study is not without limitations. Its retrospective nature may introduce biases that could affect the generalizability of the findings. Additionally, the very small sample size and the number of lymphography's included could potentially skew some outcome results. The variability in patient conditions, while reflective of clinical reality, poses challenges in deriving definitive conclusions.

Another important aspect discussed is the comparison of different management strategies for lymphatic leaks. Although no direct comparison was made between intranodal ethiodized oil injection and other techniques such as intranodal glue injection or redo surgery, the outcomes observed in this study suggest potential benefits of this approach, though limited by small sample size. The absence of clinically relevant adverse events further reinforces the argument for its safety and efficacy. Nonetheless, it is crucial to consider the potential for subclinical damage, particularly to lung parenchyma, as routine thoracic CT or lung function tests were not performed after lymphangiography (34, 37).

Given these insights and considerations, future research should focus on prospective randomized trials to validate the effectiveness of lymphangiography and refine treatment protocols. Such studies are essential for confirming the true impact of lymphangiography on chylous leakage management and could lead to significant advancements in clinical practice. This study illustrates that, in selected cases, intranodal Lipiodol® injection without embolic agents or microcatheters may be sufficient to achieve leak closure. The simplified technical approach, using only a 22G intravenous catheter, may increase accessibility to this technique in non-tertiary centers.

This study advocates for the integration of lymphangiography, specifically intranodal ethiodized oil injection, into the early treatment strategy for chylous leakage following abdominal surgery. The therapeutic benefit of Lipiodol® is thought to result from both its viscosity, which impedes lymphatic flow, and its mild sclerosing effect, which may induce inflammatory closure of disrupted vessels. The encouraging results in terms of reduced treatment duration and high success rates, along with the procedure's safety profile, position lymphangiography as a crucial element in the management of postoperative lymphatic complications. The insights gained not only contribute to a better understanding of factors influencing chylous leakage development but also pave the way for more effective and patient-centered approaches to its management.

## 5 Conclusion

This study highlights the dual role of Lipiodol® lymphangiography as both a diagnostic and therapeutic modality in the management of post-operative chylous ascites (6, 19, 27, 29). By providing precise anatomical visualization of lymphatic leaks and leveraging its inherent embolization effect, this technique offers a minimally invasive, effective, and safe alternative to conventional surgical interventions. Our findings demonstrate a 100% success rate in resolving chylous ascites with no associated complications, underscoring its potential as a first-line intervention for refractory cases.

Furthermore, this study emphasizes the importance of integrating diagnostic and therapeutic strategies into a cohesive treatment approach for complex lymphatic disorders. The ability of lymphangiography to address both the identification and resolution of chyle leaks makes it a cornerstone of modern interventional radiology. Collaboration between surgical and interventional radiology teams is essential to further optimize patient outcomes in the management of lymphatic complications.

Given the limited sample size and retrospective design of our study, future research should focus on prospective trials to validate these findings and refine protocols. Such studies are essential to establish evidence-based guidelines, optimizing the role of lymphangiography in managing chylous ascites and other lymphatic complications.

## Data Availability

The raw data supporting the conclusions of this article will be made available by the authors, without undue reservation.
